# Robust *H*_*∞*_ Filtering for a Class of Complex Networks with Stochastic Packet Dropouts and Time Delays

**DOI:** 10.1155/2014/560234

**Published:** 2014-03-27

**Authors:** Jie Zhang, Ming Lyu, Hamid Reza Karimi, Pengfei Guo, Yuming Bo

**Affiliations:** ^1^School of Automation, Nanjing University of Science & Technology, Nanjing 210094, China; ^2^Information Overall Department, North Information Control Group Co., Ltd., Nanjing 211153, China; ^3^Department of Engineering, Faculty of Engineering and Science, University of Agder, 4898 Grimstad, Norway

## Abstract

The robust *H*_*∞*_ filtering problem is investigated for a class of complex network systems which has stochastic packet dropouts and time delays, combined with disturbance inputs. The packet dropout phenomenon occurs in a random way and the occurrence probability for each measurement output node is governed by an individual random variable. Besides, the time delay phenomenon is assumed to occur in a nonlinear vector-valued function. We aim to design a filter such that the estimation error converges to zero exponentially in the mean square, while the disturbance rejection attenuation is constrained to a given level by means of the *H*_*∞*_ performance index. By constructing the proper Lyapunov-Krasovskii functional, we acquire sufficient conditions to guarantee the stability of the state detection observer for the discrete systems, and the observer gain is also derived by solving linear matrix inequalities. Finally, an illustrative example is provided to show the usefulness and effectiveness of the proposed design method.

## 1. Introduction

Over the past decades, the *H*_*∞*_ filtering problem has drawn particular attention, since *H*_*∞*_ filters are insensitive to the exact knowledge of the statistics of the noise signals. Up to now, a great deal of effort has been devoted to the design issues of various kinds of filters, for example, the Kalman filters [1–3] and *H*_*∞*_ filters [4–9].

In real-world applications, the measurements may contain missing measurements (or incomplete observations) due to various reasons such as high maneuverability of the tracked targets, sensor temporal failures or network congestion. In the past few years, the filtering problem with missing measurements has received much attention [10–17]. In [10], a model of multiple missing measurements has been presented by using a diagonal matrix to account for the different missing probabilities for individual sensors. The finite-horizon robust filtering problem has been considered in [11] for discrete-time stochastic systems with probabilistic missing measurements subject to norm-bounded parameter uncertainties. A Markovian jumping process has been employed in [12] to reflect the measurement missing problem. Moreover, the optimal filter design problem has been tackled in [13] for systems with multiple packet dropouts by solving a recursive difference equation (RDE).

On the other hand, the complex networks have been gaining increasing research attention from all fields of the basic science and the technological practice. They have applications in many real-world systems such as the Internet, World Wide Web, food webs, electric power grids, cellular and metabolic networks, scientific citation networks, and social networks [18–25]. Due to randomly occurring incomplete phenomenon which occurs in the signal transfer within complex networks, there may be time delays and packet dropouts [26–33]. For instance, over a finite horizon, the synchronization and state estimation problems for an array of coupled discrete time-varying stochastic complex networks have been studied based on the recursive linear matrix inequalities (RLMIs) approach [26]. In [29], one of the first few attempts has been made to address the synchronization problem for stochastic discrete-time complex networks with time delays. Furthermore, in [31], a new array of coupled delayed complex networks with stochastic nonlinearities, multiple stochastic disturbances, and mixed time delays in the discrete-time domain has been investigated, and the synchronization stability criteria have been derived by utilizing a novel matrix functional, the properties of the Kronecker product, the free-weighting matrix method, and the stochastic techniques.

Summarizing the above discussion, it should be pointed out that, up to now, the general filter results for complex networks with randomly occurring incomplete information have been very few, especially when the networks exhibit both stochastic natures and disturbance inputs. In this paper, we make an attempt to investigate the problems of the robust *H*_*∞*_ filtering for a class of complex systems with stochastic packet dropouts, time delays, and disturbance inputs. By constructing the proper Lyapunov-Krasovskii functional, we can get sufficient conditions, such that the filter error is exponentially stable in mean-square sense, and acquire gain of the designed observer.

The rest of the paper is organized as follows. In Section 2, the problem of complex networks is formulated and some useful lemmas are introduced. In Section 3, some sufficient conditions are established to make sure the robustly exponential stability of the filtering error dynamics. Besides, the gain of observer is also designed by LMI. An illustrated example is given in Section 4 to demonstrate the effectiveness of the proposed method. Finally, we give our conclusions in Section 5.

*Notation.* The notation used here is fairly standard except where otherwise stated. ℝ^*n*^ and ℝ^*n*×*m*^ denote, respectively, the *n* dimensional Euclidean space and the set of all *n* × *m* real matrices. *I* denotes the identity matrix of compatible dimension. The notation *X* ≥ *Y* (resp., *X* > *Y*), where *X* and *Y* are symmetric matrices, means that *X* − *Y* is positive semidefinite (resp., positive definite). *A* ^*T*^ represents the transpose of *A*. *λ*_max⁡_(*A*) and *λ*_min⁡_(*A*) denote the maximum and minimum eigenvalue of *A*, respectively. Sym{*A*} denotes the symmetric matrix *A* + *A* ^*T*^. *𝔼*{*x*} stands for the expectation of the stochastic variable *x*. ||*x*|| describes the Euclidean norm of a vector *x*. diag⁡{*F*_1_, *F*_2_,…} stands for a block-diagonal matrix whose diagonal blocks are given by *F*_1_, *F*_2_,…. The symbol ∗ in a matrix means that the corresponding term of the matrix can be obtained by symmetric property. The symbol ⊗ denotes the Kronecker product. In symmetric block matrices, the symbol ∗ is used as an ellipsis for terms induced by symmetry.

## 2. Problem Formulation

Consider the following discrete-time complex system with time delays and disturbance:

(1) xi(k+1)=f(xi(k))+g(xi(k−d(k))) +∑j=1NwijΓxj(k)+D1iv1(k)+h(xi(k))ω(k),zi(k)=Mxi(k),xi(j)=φi(j), j=−d¯M,−d¯M+1,…,0; i=1,2,…,N,

where *x*_*i*_(*k*) ∈ ℝ^*n*^ is the state vector of the *i*th node, *z*_*i*_(*k*) ∈ ℝ^*r*^ is the output of the *i*th node, *d*(*k*) denotes time-varying delay, *f*(·) and *g*(·) are nonlinear vector-valued functions satisfying certain conditions given later, *v*_1_(*k*) is the disturbance input belonging to *l*_2_([0, *∞*); ℝ^*q*^), *ω*(*k*) is a zero mean Gaussian white noise sequence, and *h*(·) is the continuous function quantifying the noise intensity. Γ = diag⁡{*r*_1_, *r*_2_,…, *r*_*n*_} is the matrix linking the *j*th state variable if *r*_*j*_ ≠ 0, and *W* = (*w*_*ij*_)_*N*×*N*_ is the coupled configuration matrix of the network with *w*_*ij*_ > 0  (*i* ≠ *j*) but not all zero. As usual, the coupling configuration matrix *W* is symmetric (i.e., *W* = *W* ^*T*^) and satisfies

(2) $$\begin{array}{c} \overset{N}{\underset{j=1}{\sum }}w_{ij}=\overset{N}{\underset{j=1}{\sum }}w_{ji}=0,\;i=1,2,\ldots ,N. \end{array}$$

*D*_1*i*_, *F*_*i*_, and *M* are constant matrices with appropriate dimensions, and *φ*_*i*_(*j*) is a given initial condition sequence.

For the system shown in (2), we make the following assumptions throughout the paper.

**Assumption 1**

The variable *ω*(*k*) is a scalar Wiener process (Brownian motion) satisfying

(3) $$\begin{array}{c} 𝔼\left\{\omega \left(k\right)\right\}=0,\;\;𝔼\left\{\omega^{2}\left(k\right)\right\}=1,\\ 𝔼\left\{\omega \left(k\right)\omega \left(j\right)\right\}=0\;\left(k\neq j\right). \end{array}$$

**Assumption 2**

The variable *d*(*k*) denotes the time-varying delay satisfying

(4) $$\begin{array}{c} 0<{\bar{d}}_{m}\leq d\left(k\right)\leq {\bar{d}}_{M}, \end{array}$$

where ${\bar{d}}_{m}$ and ${\bar{d}}_{M}$ are constant positive integers representing the lower and upper bounds on the communication delay, respectively.

**Assumption 3**

*f*(·) and *g*(·) are the nonlinear disturbance which satisfies the following sector-bounded conditions:

(5) [f(x)−f(y)−ϕ1f(x−y)]T  ×[f(x)−f(y)−ϕ2f(x−y)]≤0,[g(x)−g(y)−ϕ1g(x−y)]T  ×[g(x)−g(y)−ϕ2g(x−y)]≤0,

for all *x*, *y* ∈ ℝ^*n*^, where *ϕ*_1_^*f*^, *ϕ*_2_^*f*^, *ϕ*_1_^*g*^, and *ϕ*_2_^*g*^ are real matrices of appropriate dimensions and *f*(0) = 0,  *g*(0) = 0.

**Assumption 4**

The continuous function *h*(*x*_*i*_(*k*)) satisfies

(6) $$\begin{array}{c} h^{T}\left(x_{i}\left(k\right)\right)h\left(x_{i}\left(k\right)\right)\leq \varrho x_{i}^{T}\left(k\right)x_{i}\left(k\right), \end{array}$$

where *ϱ* > 0 is known constant scalars.

In this paper, we assume that an unreliable network medium is present between the physical plant and the state detection filter, and this means that the output data is subject to randomly missing phenomenon. The signal received by the state detection filter can be described by

(7) $$\begin{array}{c} y_{i}\left(k\right)=\alpha_{i}\left(k\right)Cx_{i}\left(k\right)+D_{2i}v_{2}\left(k\right), \end{array}$$

where *y*_*i*_(*k*) ∈ ℝ^*m*^ is the measurement output of the *i*th node and *v*_2_(*k*) is the disturbance input which belongs to *l*_2_([0, *∞*); ℝ^*p*^). *C* and *D*_2*i*_ are constant matrices with appropriate dimensions. *α*_*i*_(*k*) is the Bernoulli distributed white sequences governed by

(8) Prob{αi(k)=1}=𝔼{αi(k)}=αi,when  data  received;Prob{αi(k)=0}=1−𝔼{αi(k)}=1−αi,when  data  missing,

where *α*_*i*_ ∈ [0,1] is known constant.

In this paper, we are interested in obtaining ${\hat{z}}_{i}(k)$, the estimate of the signal *z*_*i*_(*k*), from the actual measured output *y*_*i*_(*k*). We adopt the following filter to be considered for node *i*:

(9) x^i(k+1)=f(x^i(k))+g(x^i(k−d(k))) +Ki(yi(k)−Cx^i(k)),z^i(k)=Mx^i(k),x^i(j)=0, j=−d¯M,−d¯M+1,…,0;       i=1,2,…,N,

where ${\hat{x}}_{i}(k)\in {\mathbb{R}}^{n}$ is the estimate of the state *x*_*i*_(*k*), ${\hat{z}}_{i}(k)\in {\mathbb{R}}^{r}$ is the estimate of the output *z*_*i*_(*k*), and *K*_*i*_ ∈ ℝ^*n*×*m*^ is the estimator gain matrix to be designed.

Let the estimation error be $e(k)=x(k)-\hat{x}(k)$. By using the Kronecker product, the filtering error system can be obtained from (2), (7), and (9) as follows:

(10) ek+1=f~k+g~k−dk−KC~ek+(W⊗Γ+KC~)xk +L~vk+hkωk−K(∑i=1Nαi(k)EiC~xk),z~k=M~ek,

where

(11) $$\begin{array}{c} x_{k}={\left[\begin{array}{cccc} x_{1}^{T}\left(k\right) & x_{2}^{T}\left(k\right) & \cdots & x_{N}^{T}\left(k\right) \end{array}\right]}^{T},\\ {\hat{x}}_{k}={\left[\begin{array}{cccc} {\hat{x}}_{1}^{T}\left(k\right) & {\hat{x}}_{2}^{T}\left(k\right) & \cdots & {\hat{x}}_{N}^{T}\left(k\right) \end{array}\right]}^{T},\\ z_{k}={\left[\begin{array}{cccc} z_{1}^{T}\left(k\right) & z_{2}^{T}\left(k\right) & \cdots & z_{N}^{T}\left(k\right) \end{array}\right]}^{T},\\ {\hat{z}}_{k}={\left[\begin{array}{cccc} {\hat{z}}_{1}^{T}\left(k\right) & {\hat{z}}_{2}^{T}\left(k\right) & \cdots & {\hat{z}}_{N}^{T}\left(k\right) \end{array}\right]}^{T},\;\;{\tilde{z}}_{k}=z_{k}-{\hat{z}}_{k},\\ v_{k}={\left[\begin{array}{cc} v_{1}^{T}\left(k\right) & v_{2}^{T}\left(k\right) \end{array}\right]}^{T},\;\;d_{k}=d\left(k\right),\;\;w_{k}=w\left(k\right),\\ f\left(x_{k}\right)={\left[\begin{array}{cccc} f^{T}\left(x_{1}\left(k\right)\right) & f^{T}\left(x_{2}\left(k\right)\right) & \cdots & f^{T}\left(x_{N}\left(k\right)\right) \end{array}\right]}^{T},\\ g\left(x_{k}\right)={\left[\begin{array}{cccc} g^{T}\left(x_{1}\left(k\right)\right) & g^{T}\left(x_{2}\left(k\right)\right) & \cdots & g^{T}\left(x_{N}\left(k\right)\right) \end{array}\right]}^{T},\\ h\left(x_{k}\right)={\left[\begin{array}{cccc} h^{T}\left(x_{1}\left(k\right)\right) & h^{T}\left(x_{2}\left(k\right)\right) & \cdots & h^{T}\left(x_{N}\left(k\right)\right) \end{array}\right]}^{T},\\ {\tilde{f}}_{k}=f\left(x_{k}\right)-f\left({\hat{x}}_{k}\right),\;\;{\tilde{g}}_{k}=g\left(x_{k}\right)-g\left({\hat{x}}_{k}\right),\\ K=\;\mathrm{diag}\left\{K_{1},K_{2},\ldots ,K_{N}\right\},\;\;\tilde{C}=I⊗C,\\ D_{1}={\left[\begin{array}{cccc} D_{11}^{T} & D_{12}^{T} & \cdots & D_{1N}^{T} \end{array}\right]}^{T},\;\;\tilde{D}=\left[\begin{array}{cc} D_{1} & -KD_{2} \end{array}\right],\\ D_{2}={\left[\begin{array}{cccc} D_{21}^{T} & D_{22}^{T} & \cdots & D_{2N}^{T} \end{array}\right]}^{T},\;\;\tilde{M}=I⊗M,\\ E_{i}=\;\mathrm{diag}\left\{\underset{i-1}{\underset{︸}{0,\ldots ,0}},I,\underset{N-i}{\underset{︸}{0,\ldots ,0}}\right\}. \end{array}$$

Setting $\eta_{k}={\left[\begin{array}{cc} x_{k}^{T} & e_{k}^{T} \end{array}\right]}^{T}$, we subsequently obtain an augmented system as follows:

(12) ηk+1=𝒲ηk+f→k+g→k−dk +∑i=1N(αki−αi)𝒢iC~𝒮ηk+𝒟vk+ℋωk,z~k=ℳηk,

where

(13) $$\begin{array}{c} {\vec{f}}_{k}={\left[\begin{array}{cc} f^{T}\left(x_{k}\right) & {\tilde{f}}_{k}^{T} \end{array}\right]}^{T},\;\;{\vec{g}}_{k}={\left[\begin{array}{cc} g^{T}\left(x_{k}\right) & {\tilde{g}}_{k}^{T} \end{array}\right]}^{T},\\ \alpha_{k}^{i}=\alpha_{i}\left(k\right),\;\;{\tilde{\alpha }}^{\Lambda }=\;\mathrm{diag}\left\{\alpha_{1}I,\alpha_{2}I,\ldots ,\alpha_{N}I\right\},\\ {𝒢}_{i}={\left[\begin{array}{cc} 0 & -E_{i}^{T}K^{T} \end{array}\right]}^{T},\\ ℳ=\left[\begin{array}{cc} 0 & \tilde{M} \end{array}\right],\;\;𝒮=\left[\begin{array}{cc} I & 0 \end{array}\right],\\ 𝒲=\left[\begin{array}{cc} W⊗\Gamma & 0\\ W⊗\Gamma +K\left(I-{\tilde{\alpha }}^{\Lambda }\right)\tilde{C} & -K\tilde{C} \end{array}\right],\\ 𝒟=\left[\begin{array}{cc} D_{1} & 0\\ D_{1} & -KD_{2} \end{array}\right],\;\;ℋ=\left[\begin{array}{c} h\left(x_{k}\right)\\ h\left(x_{k}\right) \end{array}\right]. \end{array}$$

**Definition 5 (see [34])**

The filtering error system (12) is said to be exponentially stable in the mean square if, in case of *v*_*k*_ = 0, for any initial conditions, there exist constants *ε* > 0 and 0 < *κ* < 1 such that

(14) $$\begin{array}{c} 𝔼\left\{{||\eta_{k}||}^{2}\right\}\leq \varepsilon \kappa^{k}\;\underset{i\in [-{\bar{d}}_{M},\;0]}{\max}\;𝔼\left\{{||\eta_{i}||}^{2}\right\},\;k\in \mathbb{N}, \end{array}$$

where *η*_*i*_ : = [*φ*_1_^*T*^(*i*), *φ*_2_^*T*^(*i*),…, *φ*_*N*_^*T*^(*i*), *φ*_1_^*T*^(*i*), *φ*_2_^*T*^(*i*),…, *φ*_*N*_^*T*^(*i*)]^*T*^, for all $i\in [-{\bar{d}}_{M},0]$.

Our aim in this paper is to develop techniques to deal with the robust *H*_*∞*_ filtering problem for a class of complex systems with stochastic packet dropouts, time delays, and disturbance inputs. The augmented observer system (12) satisfies the following requirements (Q1) and (Q2), simultaneously:

(Q1) the filter error system (12) with *v*_*k*_ = 0 is exponentially stable in the mean square;
(Q2) under the zero initial condition, the filtering error ${\tilde{z}}_{k}$ satisfies
  (15) $$\begin{array}{c} \frac{1}{N}\overset{\infty }{\underset{k=0}{\sum }}𝔼\left\{{||{\tilde{z}}_{k}||}^{2}\right\}\leq \gamma^{2}\overset{\infty }{\underset{k=0}{\sum }}{||v_{k}||}^{2} \end{array}$$

for all nonzero *v*_*k*_, where *γ* > 0 is a given disturbance attenuation level.

**Lemma 6 (the Schur complement)**

Given constant matrices *S*_1_, *S*_2_,  and  *S*_3_, where *S*_1_ = *S*_1_^*T*^ and 0 < *S*_2_ = *S*_2_^*T*^, then *S*_1_ + *S*_3_^*T*^ *S*_2_^−1^ *S*_3_ < 0 if and only if

(16) $$\begin{array}{c} \left[\begin{array}{cc} S_{1} & S_{3}^{T}\\ S_{3} & -S_{2} \end{array}\right]<0\;\text{or}\;\left[\begin{array}{cc} -S_{2} & S_{3}\\ S_{3}^{T} & S_{1} \end{array}\right]<0. \end{array}$$

## 3. Main Results

In this part, we will construct the Lyapunov-Krasovskii functional and the use of linear matrix inequality to propose sufficient conditions such that the system error model in (12) could be exponentially stable in mean square. Let us first consider the robust exponential stability analysis problem for the filter error system (12) with *v*_*k*_ = 0.

**Theorem 7**

Consider the system (2) and suppose that the estimator parameters *K*_*i*_ (*i* = 1,2,…, *N*) are given. The system augmented error model (12) with *v*_*k*_ = 0 is said to be exponentially stable in mean square, if there exist positive definite matrices *Q*_*i*_  (*i* = 1,2, 3,4) and positive scalars *λ*_*j*_  (*j* = 1,2, 3) satisfying the following inequality:

(17) $$\begin{array}{c} \Pi_{1}=\left[\begin{array}{cccc} \Xi_{11} & 0 & \Xi_{13} & {𝒲}^{T}P_{1}\\ {}* & \Xi_{22} & 0 & \lambda_{2}\Phi_{2}^{gT}\\ {}* & * & \Xi_{33} & P_{1}\\ {}* & * & * & \Xi_{44} \end{array}\right]<0,\\ P_{1}\leq \lambda_{3}I, \end{array}$$

where

(18) α~i∗=αi(1−αi),  Aϱ=[ϱI000],Φ1f=I⊗
Sym
{12ϕ1fTϕ2f},Φ2f=I⊗(ϕ1f+ϕ2f)2,Φ1g=I⊗
Sym
{12ϕ1gTϕ2g},Φ2g=I⊗(ϕ1g+ϕ2g)2,P1=diag⁡{I⊗Q1,I⊗Q2},  Ξ22=−P2−λ2Φ1g,P2=diag⁡{I⊗Q3,I⊗Q4},  Ξ33=P1−λ1I,Ξ11=𝒲TP1𝒲−P1+(d¯M−d¯m+1)P2+λ3Aϱ −λ1Φ1f+∑i=1Nα~i∗𝒮TC~T𝒢iTP1𝒢iC~𝒮,Ξ13=𝒲TP1+λ1Φ2fT,  Ξ44=P1−λ2I.

**Proof**

Choose the following Lyapunov functional for system (12):

(19) $$\begin{array}{c} V\left(k\right)=V_{1}\left(k\right)+V_{2}\left(k\right)+V_{3}\left(k\right), \end{array}$$

where

(20) $$\begin{array}{c} V_{1}\left(k\right)=\eta_{k}^{T}P_{1}\eta_{k},\\ V_{2}\left(k\right)=\overset{k-1}{\underset{i=k-d_{k}}{\sum }}\eta_{i}^{T}P_{2}\eta_{i},\\ V_{3}\left(k\right)=\overset{k-{\bar{d}}_{m}}{\underset{j=k-{\bar{d}}_{M}+1}{\sum }}\;‍\overset{k-1}{\underset{i=j}{\sum }}\eta_{i}^{T}P_{2}\eta_{i}. \end{array}$$

Then, along the trajectory of system (12) with *v*_*k*_ = 0, we have

(21) 𝔼{ΔV1(k)}=𝔼{V1(k+1)−V1(k)}=𝔼{ηk+1TP1ηk+1−ηkTP1ηk}=𝔼{ηkT𝒲TP1𝒲ηk+f→kTP1f→k  +g→k−dkTP1g→k−dk+ℋTP1ℋ  +∑i=1Nα~i∗ηkT𝒮TC~T𝒢iTP1𝒢iC~𝒮ηk  +2ηkT𝒲TP1f→k+2ηkT𝒲TP1g→k−dk  +2f→kTP1g→k−dk−ηkTP1ηk},𝔼{ℋTP1ℋ}≤λ3ηkTAϱηk.

Next, it can be derived that

(22) 𝔼{ΔV2(k)}=𝔼{V2(k+1)−V2(k)}=𝔼{∑i=k−dk+1+1k ηiTP2ηi−∑i=k−dkk−1ηiTP2ηi}=𝔼{ηkTP2ηk−ηk−dkTP2ηk−dk   +∑i=k−dk+1+1k−1 ηiTP2ηi−∑i=k−dk+1k−1ηiTP2ηi}=𝔼{ηkTP2ηk−ηk−dkTP2ηk−dk+∑i=k−d¯m+1k−1 ηiTP2ηi   +∑i=k−dk+1+1k−d¯m ‍ηiTP2ηi−∑i=k−dk+1k−1ηiTP2ηi}≤𝔼{ηkTP2ηk−ηk−dkTP2ηk−dk   +∑i=k−d¯M+1k−d¯m ηiTP2ηi},𝔼{ΔV3(k)} =𝔼{V3(k+1)−V3(k)} =𝔼{∑j=k−d¯M+2k−d¯m+1 ‍∑i=jkηiTP2ηi−∑j=k−d¯M+1k−d¯m ‍∑i=jk−1ηiTP2ηi} =𝔼{∑j=k−d¯M+1k−d¯m ∑i=j+1kηiTP2ηi−∑j=k−d¯M+1k−d¯m ‍∑i=jk−1ηiTP2ηi} =𝔼{∑j=k−d¯M+1k−d¯m(ηkTP2ηk−ηjTP2ηj)} =𝔼{(d¯M−d¯m)ηkTP2ηk−∑i=k−d¯M+1k−d¯mηiTP2ηi}.

Letting

(23) $$\begin{array}{c} \xi_{k}={\left[\begin{array}{cccc} \eta_{k}^{T} & \eta_{k-d_{k}}^{T} & {\vec{f}}_{k}^{T} & \;{\vec{g}}_{k-d_{k}}^{T} \end{array}\right]}^{T}, \end{array}$$

the combination of (21) and (22) results in

(24) 𝔼{ΔV(ηk)}=𝔼{V(k+1)−V(k)}=∑i=13𝔼{ΔVi(k)}≤𝔼{ξkTΠ~1ξk},

where

(25) Π~1=[Ξ~110𝒲TP1𝒲TP1∗−P200∗∗P1P1∗∗∗P1],Ξ~11=𝒲TP1𝒲−P1+(d¯M−d¯m+1)P2 +λ3Aϱ+∑i=1Nα~i∗𝒮TC~T𝒢iTP1𝒢iC~𝒮,

Notice that (5) implies

(26) $$\begin{array}{c} {\left[{\vec{f}}_{k}-\left(I⊗\phi_{1}^{f}\right)\eta_{k}\right]}^{T}\left[{\vec{f}}_{k}-\left(I⊗\phi_{2}^{f}\right)\eta_{k}\right]\leq 0.\\ {\left[{\vec{g}}_{k}-\left(I⊗\phi_{1}^{g}\right)\eta_{k}\right]}^{T}\left[{\vec{g}}_{k}-\left(I⊗\phi_{2}^{g}\right)\eta_{k}\right]\leq 0. \end{array}$$

From (26), it follows that

(27) 𝔼{ΔV(ηk)}  ≤𝔼{ξkTΠ~1ξk−λ1[f→k−(I⊗ϕ1f)ηk]T     ×[f→k−(I⊗ϕ2f)ηk]     −λ2[g→k−dk−(I⊗ϕ1g)ηk−dk]T     ×[g→k−dk−(I⊗ϕ2g)ηk−dk]}  ≤𝔼{ξkTΠ1ξk}.

According to Theorem 7, we have Π_1_ < 0; there must exist a sufficiently small scalar *ε*_0_ > 0 such that

(28) $$\begin{array}{c} \Pi_{1}+\varepsilon_{0}\mathrm{diag}\left\{I,0\right\}<0. \end{array}$$

Then, it is easy to see from (27) and (28) that the following inequality holds:

(29) $$\begin{array}{c} 𝔼\left\{\Delta V\left(\eta_{k}\right)\right\}\leq -\varepsilon_{0}𝔼\left\{{||\eta_{k}||}^{2}\right\}. \end{array}$$

According to the definition of *V*(*k*), we can derive that

(30) $$\begin{array}{c} 𝔼\left\{V\left(k\right)\right\}\leq \rho_{1}𝔼\left\{{||\eta_{k}||}^{2}\right\}+\rho_{2}\overset{k-1}{\underset{i=k-{\bar{d}}_{M}}{\sum }}𝔼\left\{{||\eta_{i}||}^{2}\right\}, \end{array}$$

where *ρ*_1_ = *λ*_max⁡_(*P*_1_) and $\rho_{2}=({\bar{d}}_{M}-{\bar{d}}_{m}+1)\lambda_{\max}(P_{2})$.

For any scalar *μ* > 1, together with (19), the above inequality implies that

(31) μk+1𝔼{V(k+1)}−μk𝔼{V(k)} =μk+1𝔼{ΔV(k)}+μk(μ−1)𝔼{V(k)} ≤ϵ1(μ)μk𝔼{||ηk||2}+ϵ2(μ)∑i=k−d¯Mk−1μk𝔼{||ηi||2}

with *ϵ*_1_(*μ*) = (*μ* − 1)*ρ*_1_ − *με*_0_ and *ϵ*_2_(*μ*) = (*μ* − 1)*ρ*_2_.

In addition, for any integer $m\geq {\bar{d}}_{M}+1$, summing up both sides of (31) from 0 to *m* − 1 with respect to *k*, we have

(32) μm𝔼{V(k+1)}−𝔼{V(0)}  ≤ϵ1(μ)∑k=0m−1μk𝔼{||ηk||2}   +ϵ2(μ)∑k=0m−1  ∑i=k−d¯Mk−1μk𝔼{||ηi||2}.

Due to ${\bar{d}}_{M}\geq 1$,

(33) ∑k=0m−1  ∑i=k−d¯Mk−1μk𝔼{||ηi||2}  ≤(∑i=−d¯M−1 ∑k=0i+d¯M+∑i=0m−1−d¯M ∑k=i+1i+d¯M    +∑i=m−1−d¯Mm−1 ∑k=i+1m−1)μk𝔼{||ηi||2}  ≤d¯M∑i=−d¯M−1μi+d¯M𝔼{||ηi||2}   +d¯M∑i=0m−1−d¯Mμi+d¯M𝔼{||ηi||2}   +d¯M∑i=m−1−d¯Mm−1μi+d¯M𝔼{||ηi||2}  ≤d¯Mμd¯Mmax⁡−d¯M≤i≤0𝔼{||ηi||2}   +d¯Mμd¯M∑i=0m−1μi𝔼{||ηi||2}.

So, we can obtain from (32) and (33) the following:

(34) μk𝔼{V(k)}≤𝔼{V(0)}+(ϵ1(μ)+ϵ¯2(μ))∑i=0k−1μi𝔼{||ηi||2} +ϵ¯2(μ)∑−d¯M≤i≤0𝔼{||ηi||2},

with ${\bar{\epsilon }}_{2}(\mu )={\bar{d}}_{M}\mu^{{\bar{d}}_{M}}(\mu -1)\rho_{2}$.

Let *ρ*_0_ = *λ*_min⁡_(*P*_1_) and *ρ* = max⁡{*ρ*_1_, *ρ*_2_}. It is obvious from (19) that

(35) $$\begin{array}{c} 𝔼\left\{V\left(k\right)\right\}\geq \rho_{0}𝔼\left\{{||\eta_{k}||}^{2}\right\}. \end{array}$$

Meanwhile, we can find easily from (30) that

(36) $$\begin{array}{c} 𝔼\left\{V\left(0\right)\right\}\leq \rho \left(2{\bar{d}}_{M}+1\right)\;\underset{-{\bar{d}}_{M}\leq i\leq 0}{\max}\;𝔼\left\{{||\eta_{i}||}^{2}\right\}. \end{array}$$

It can be verified that there exists a scalar *μ*_0_ > 1 such that

(37) $$\begin{array}{c} \epsilon_{1}\left(\mu_{0}\right)+{\bar{\epsilon }}_{2}\left(\mu_{0}\right)=0. \end{array}$$

Therefore, from (34)–(37), it is clear to see that

(38) 𝔼{||ηk||2} ≤(1μ0)kρ(2d¯M+1)+d¯Mϵ¯2(μ0)ρ0max⁡−d¯M≤i≤0 𝔼{||ηi||2}.

The augmented system (12) with *v*_*k*_ = 0 is exponentially mean-square stable according to Definition 5. The proof is complete.

Next, we will analyze the performance of the filtering error system (12).

**Theorem 8**

Consider the system (2) and suppose that the estimator parameters *K*_*i*_ (*i* = 1,2,…, *N*) are given. The system augmented error model (12) is said to be exponentially stable in mean square and satisfies the *H*_*∞*_ performance constraint (15) for all nonzero *v*_*k*_ and *ω*_*k*_ under the zero initial condition, if there exist positive definite matrices *Q*_*i*_  (*i* = 1,2, 3,4) and positive scalars *λ*_*j*_  (*j* = 1,2, 3) satisfying the following inequality:

(39) $$\begin{array}{c} \Pi_{2}=\left[\begin{array}{ccccc} \Xi_{11}^{*} & 0 & \Xi_{13} & {𝒲}^{T}P_{1} & {𝒲}^{T}P_{1}𝒟\\ {}* & \Xi_{22} & 0 & \lambda_{2}\Phi_{2}^{gT} & 0\\ {}* & * & \Xi_{33} & P_{1} & P_{1}𝒟\\ {}* & * & * & \Xi_{44} & P_{1}𝒟\\ {}* & * & * & * & {𝒟}^{T}P_{1}𝒟-\gamma^{2}I \end{array}\right]<0,\\ P_{1}\leq \lambda_{3}I, \end{array}$$

where

(40) Ξ11∗=𝒲TP1𝒲−P1+(d¯M−d¯m+1)P2 −λ1Φ1f+λ3Aϱ +1NℳTℳ+∑i=1Nα~i∗𝒮TC~T𝒢iTP1𝒢iC~𝒮,

and other parameters are defined as in Theorem 7.

**Proof**

It is clear that (39) implies (17). According to Theorem 7, the filtering error system (12) with *v*_*k*_ = 0 is robustly exponentially stable in the mean square.

Let us now deal with the performance of the system (15). Construct the same Lyapunov-Krasovskii functional as in Theorem 7. A similar calculation as in the proof of Theorem 7 leads to

(41) 𝔼{ΔV(k)}≤𝔼{ξkTΠ1ξk+2vkT𝒟TP1𝒲ηk   +2vkT𝒟TP1f→k+2vkT𝒟TP1g→k−dk   +vkT𝒟TP1𝒟vk},

where *ξ*_*k*_ and Π_1_ are defined previously.

Setting ${\tilde{\xi }}_{k}={\left[\begin{array}{cc} \xi_{k}^{T} & v_{k}^{T} \end{array}\right]}^{T}$, inequality (41) can be rewritten as

(42) $$\begin{array}{c} 𝔼\left\{\Delta V\left(k\right)\right\}\leq 𝔼\left\{{\tilde{\xi }}_{k}^{T}\left[\begin{array}{cc} \Pi_{1} & {\tilde{𝒟}}^{T}\\ {}* & {𝒟}^{T}P_{1}𝒟 \end{array}\right]{\tilde{\xi }}_{k}\right\}, \end{array}$$

where $\tilde{𝒟}=\left[\begin{array}{cccc} {𝒟}^{T}P_{1}𝒲 & 0 & {𝒟}^{T}P_{1} & {𝒟}^{T}P_{1} \end{array}\right]$.

In order to deal with the *H*_*∞*_ performance of the filtering system (12), we introduce the following index:

(43) $$\begin{array}{c} 𝒥\left(s\right)=𝔼\overset{s}{\underset{k=0}{\sum }}\left\{\frac{1}{N}{||{\tilde{z}}_{k}||}^{2}-\gamma^{2}{||v_{k}||}^{2}\right\}, \end{array}$$

where *s* is nonnegative integer.

Under the zero initial condition, one has

(44) 𝒥(s)=𝔼∑k=0s{1N||z~k||2−γ2||vk||2+ΔV(k)} −𝔼{V(s+1)}≤𝔼∑k=0s{1N||z~k||2−γ2||vk||2+ΔV(k)}≤𝔼∑k=0s{ξ~kTΠ2ξ~k}<0.

According to Theorem 8, we have *𝒥*(*s*) ≤ 0. Letting *s* → *∞*, we obtain

(45) $$\begin{array}{c} \frac{1}{N}\overset{\infty }{\underset{k=0}{\sum }}𝔼\left\{{||{\tilde{z}}_{k}||}^{2}\right\}\leq \gamma^{2}\overset{\infty }{\underset{k=0}{\sum }}{||v_{k}||}^{2}, \end{array}$$

and the proof is now complete.

We aim at solving the filter design problem for complex network (2). Therefore, we are in a position to consider the *H*_*∞*_ filter design problem for the complex network (2). The following theorem provides sufficient conditions for the existence of such filters for system (12). The following result can be easily accessible from Theorem 8, and the proof is therefore omitted.

**Theorem 9**

Consider the system (2) and suppose that the disturbance attenuation level *γ* > 0 is given. The system augmented error model (12) is said to be exponentially stable in mean square and satisfies the *H*_*∞*_ performance constraint (15) for all nonzero *v*_*k*_ and *ω*_*k*_ under the zero initial condition, if there exist positive definite matrices *Q*_*i*_  (*i* = 1,2, 3,4), matrices *Y*_*i*_ (*i* = 1,2,…, *N*), and positive scalars *λ*_*j*_  (*j* = 1,2, 3) satisfying the following inequality:

(46) $$\begin{array}{c} \Pi_{3}=\left[\begin{array}{cccccccc} \Pi_{11} & \Pi_{12} & 0 & \Pi_{14} & \Pi_{15} & \Pi_{16} & \Pi_{18} & \Pi_{19}\\ {}* & \Pi_{22} & 0 & \Pi_{24} & \Pi_{25} & \Pi_{26} & \Pi_{28} & 0\\ {}* & * & \Pi_{33} & 0 & \Pi_{35} & 0 & 0 & 0\\ {}* & * & * & \Pi_{44} & \Pi_{45} & \Pi_{46} & 0 & 0\\ {}* & * & * & * & \Pi_{55} & \Pi_{56} & 0 & 0\\ {}* & * & * & * & * & \Pi_{66} & \Pi_{68} & 0\\ {}* & * & * & * & * & * & -{𝒬}_{2} & 0\\ {}* & * & * & * & * & * & * & -{𝒬}_{2} \end{array}\right]<0,\\ P_{1}\leq \lambda_{3}I, \end{array}$$

where

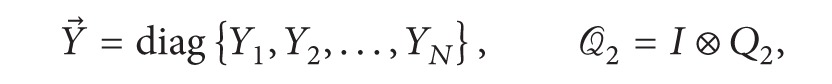
(47)

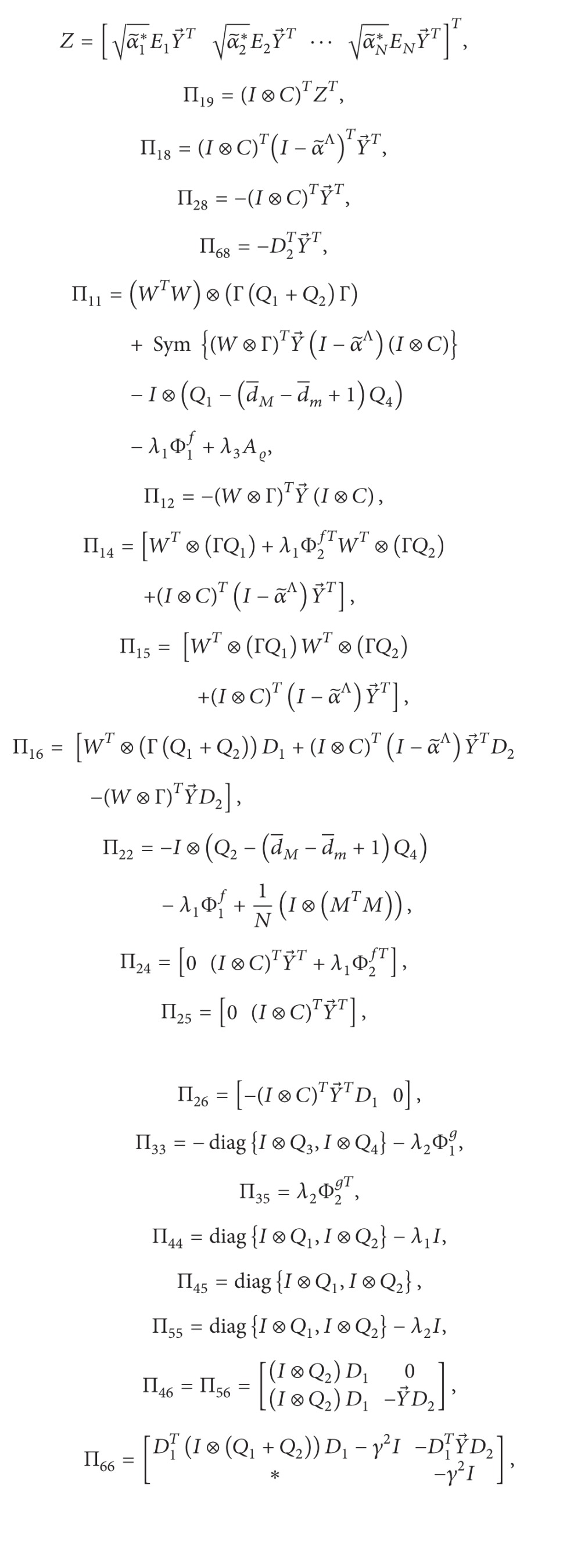
(48)
and other parameters are defined as in Theorem 7. Moreover, if the above inequality is feasible, the desired state estimator gains can be determined by

(49) $$\begin{array}{c} K_{i}=Q_{2}^{-1}Y_{i}. \end{array}$$

## 4. Numerical Simulations

In this section, we present an illustrative example to demonstrate the effectiveness of the proposed theorems. Considering the system model (2) with three sensors, the system data are given as follows:

(50) W=[−0.40.400.4−0.60.200.2−0.2],  Γ=  diag⁡{0.1,0.1},D11=[0.14−0.15],  D12=[0.10.12],D13=[0.1−0.05],  M=[0.50.7],D21=[0.1−0.1],  D22=[−0.10.2],D23=[0.2−0.15],  C=[0.80.50.9−0.3],f(xi(k)) =[−0.6xi1(k)+0.3xi2(k)+  tanh(0.3xi1(k))0.6xi2(k)−  tanh(0.2xi2(k))],g(xi(k)) =[0.02xi1(k)+0.06xi2(k)−0.03xi1(k)+0.02xi2(k)+tanh⁡(0.01xi1(k))],h(xi(k))=0.15xi(k),  d(k)=2+sin⁡(πk2),v1(k)=3exp⁡⁡(−0.3k)cos⁡⁡(0.2k),v2(k)=2exp⁡(−0.2k)sin(0.1k).

Then, it is easy to see that the constraint (26) can be met with

(51) $$\begin{array}{c} \phi_{1}^{f}=\left[\begin{array}{cc} -0.6 & 0.3\\ 0 & 0.4 \end{array}\right],\;\;\phi_{2}^{f}=\left[\begin{array}{cc} -0.3 & 0.3\\ 0 & 0.6 \end{array}\right],\\ \phi_{1}^{g}=\left[\begin{array}{cc} 0.02 & 0.06\\ -0.03 & 0.02 \end{array}\right],\;\;\phi_{2}^{g}=\left[\begin{array}{cc} 0.02 & 0.06\\ -0.02 & 0.02 \end{array}\right]. \end{array}$$

Let the disturbance attenuation level be *γ* = 0.96. Assume that the initial values *φ*_*i*_(*k*) (*i* = 1,2, 3;   *k* = −3, −2, −1,0) are generated that obey uniform distribution over [−1.5, 1.5], *α*_1_ = 0.88, *α*_2_ = 0.85, and *α*_3_ = 0.87, and the delay parameters are chosen as ${\bar{d}}_{m}=1$ and ${\bar{d}}_{M}=3$.

By applying Theorem 9 with help from MATLAB, we can obtain the desired filter parameters as follows:

(52)λ1=23.9040,  λ2=50.2256,  λ3=14.0023,Q1=[9.14463.89083.89083.5213],Q2=[9.07683.72623.72626.7169],Q3=[0.7093−0.1693−0.16930.2576],Q4=[0.8390−0.3283−0.32830.8284],Y1=[0.5154−0.92700.9371−0.6966],Y2=[0.6617−1.10421.0172−0.8178],(53)Y3=[0.2943−0.74410.8713−0.6346].

Then, according to (49), the desired estimator parameters can be designed as

(54) $$\begin{array}{c} K_{1}=\left[\begin{array}{cc} -0.0006 & -0.0771\\ 0.1399 & -0.0609 \end{array}\right],\\ K_{2}=\left[\begin{array}{cc} 0.0139 & -0.0928\\ 0.1437 & -0.0703 \end{array}\right],\\ K_{3}=\left[\begin{array}{cc} -0.0270 & -0.0559\\ 0.1447 & -0.0635 \end{array}\right]. \end{array}$$

**Figure 1:**
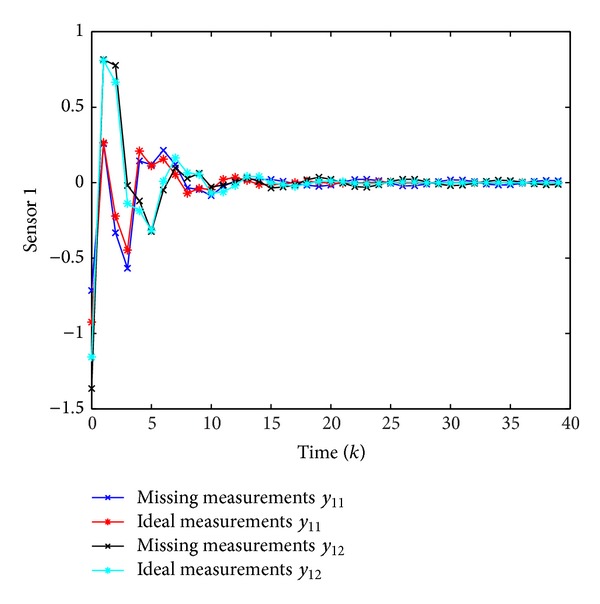
The ideal measurements and the missing measurements of *y*_1_(*k*).

**Figure 2:**
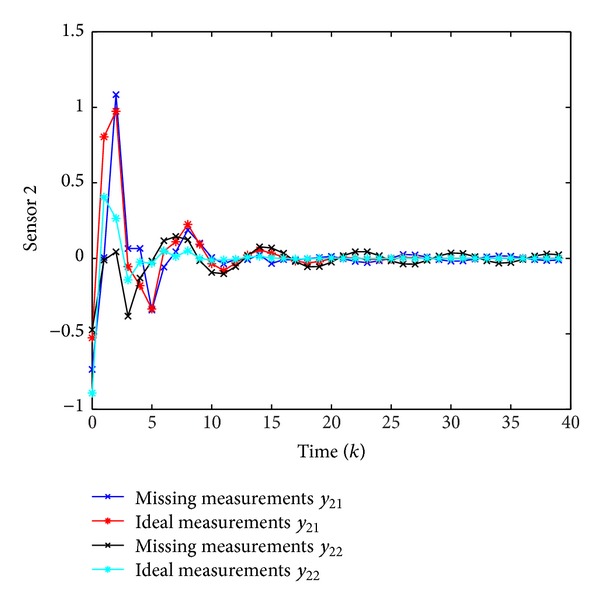
The ideal measurements and the missing measurements of *y*_2_(*k*).

**Figure 3:**
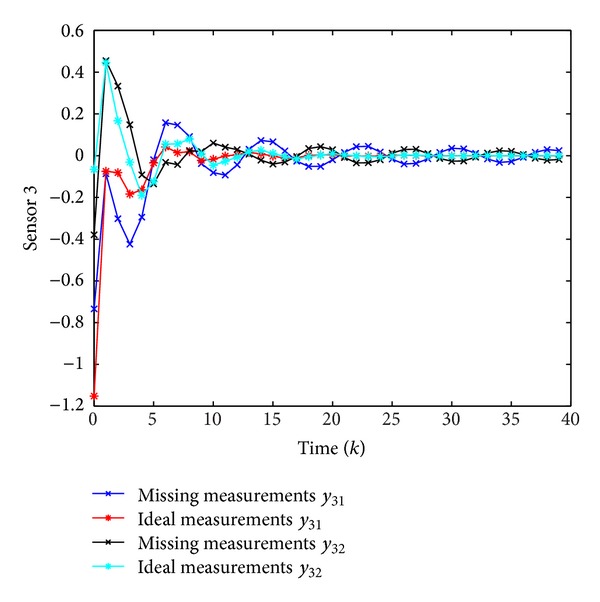
The ideal measurements and the missing measurements of *y*_3_(*k*).

**Figure 4:**
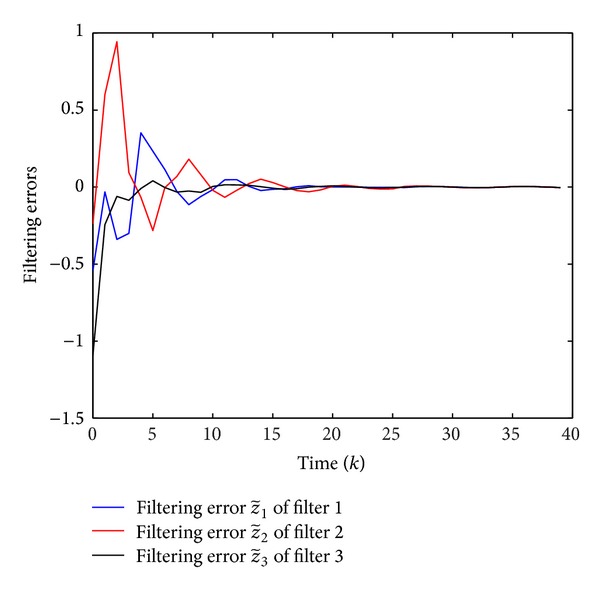
The estimator errors ${\hat{z}}_{i}(k)\;(i=1,2,3)$.

Simulation results are shown in Figures 1, 2, 3, and 4, where Figures 1–3 plot the missing measurements and ideal measurements for sensors 1–3, respectively, and Figure 4 depicts the output errors. From those figures, we can confirm the superiority of the designed *H*_*∞*_ filter.

## 5. Conclusions

In this paper, we have studied the robust *H*_*∞*_ filtering problem for a class of complex systems with stochastic packet dropouts, time delays, and disturbance inputs. The discrete-time system under study involves multiplicative noises, time-varying delays, sector-bounded nonlinearities, and stochastic packet dropouts. By means of LMIs, sufficient conditions for the robustly exponential stability of the filtering error dynamics have been obtained and, at the same time, the prescribed disturbance rejection attenuation level has been guaranteed. Then, the explicit expression of the desired filter parameters has been derived. A numerical example has been provided to show the usefulness and effectiveness of the proposed design method.
